# Surrogacy as a good option for treatment of repeated implantation failure: a case series

**Published:** 2013-01

**Authors:** Nastaran Aflatoonian, Maryam Eftekhar, Behrooz Aflatoonian, Elham Rahmani, Abass Aflatoonian

**Affiliations:** 1 *Madar Hospital, Yazd, Iran.*; 2 *Department of Obstetrics and Gynecology, Yazd Research and Clinical Center for Infertility, Shahid Sadoughi University of Medical Sciences, Yazd, Iran.*; 3 *Department of Obstetrics and Gynecology, Bushehr University of Medical Sciences, Bushehr, Iran.*

**Keywords:** *Repeated implantation failure*, *Surrogacy*, *In vitro fertilization*, *Pregnancy rate*

## Abstract

**Background: **Repeated implantation failure (RIF) is defined as pregnancy failure after two to six times with at least ten high grade embryo transfer to uterus. A variety of causes have been anticipated for RIF, including anatomical, autoimmune, genetics, endocrine and thrombotic anomalies. Factors responsible for RIF have important implication regarding treatment however in many couples a perfect cause cannot be found.

**Cases:** In these case series, we reported nine couples with RIF that after investigation no definitive etiology was found for RIF and empirical therapy by heparin, aspirin and or immunotherapy was not effective. In these cases we recommended transfer of embryos to surrogate uterus. Nine patients were studied and six of them developed a normal pregnancy (pregnancy rate=66.66%).

**Conclusion:** This study showed that surrogacy is a good option for treatment of RIF.

## Introduction

Assisted reproductive technology has been progressed during recent years (1, 2). Despite of the introduction of enhanced protocols for ovarian stimulation and improved culturing techniques for embryo development, it has been estimated that live birth-rate per cycle is ranged from 29.9-43.7% (3). Repeated implantation failure (RIF) was defined as pregnancy failure after two to six times with at least ten high grade embryo transfer to uterus (1). 

Successful implantation requires a receptive endometrium, a good embryo and synchronization between endometrium and embryos. The endometrium undergoes a series of proliferative and secretory alterations, and endometrial receptivity exist in a short time of cycles, recognized as the “implantation window”    (4) . Endometrial development needs the perfect association of a really large number of different aspects. Although our information of endometrial receptivity is incomplete, it may still allow for important development in the organization of female infertility    (5) . 

A variety of causes have been anticipated for RIF, including anatomical, autoimmune, genetics, endocrine and thrombotic anomalies (6, 7). Recent evidence suggested that these different abnormalities increase levels of circulating natural killer cells and interfere with implantation (8). Factors responsible for RIF have important implication regarding treatment however in many couples a definitive cause cannot be found (9). Although many molecules concerned in implantation have been recognized in humans, microarray analysis of the endometrium has only presented an insight into the role of a few molecules (5). 

The molecular mechanisms which control endometrial-blastocyst interaction remain poorly clear. In this case series, we reported a series of couple with RIF that after investigation no definitive etiology was found for RIF and empirical therapy by heparin, aspirin and or immunotherapy was not effective. In these cases In order to exclude intrauterine factors that might impede embryo implantation, we recommended transfer of embryos to surrogate uterus.

## Case Series

We reported a case series of patients with history of RIF at Yazd Research and Clinical Center for Infertility of Shahid Sadoughi University of Medical Sciences and Yazd Madar Hospital, from January 2009 to December 2011. A signed informed consent was obtained from all of the patients who participated in the study. Basic demographic characteristic of patients is presented in table I. All of the couples had history of transfer of high quality embryos in previous cycles. Evaluation of uterine cavity by vaginal sonography, hysteroscopy and/or sonohysterography showed normal uterine cavity. Lupus anticoagulant, anti-cardiolipin antibodies, protein S and protein C were measured in all of the cases and the values were reported in normal ranges. Karyotypes of all couples were normal. 

Females were between 27-43 years old. According to the request of couples, we suggested transferring the obtained embryos to a surrogate’s uterus. Less than three embryos were transferred. All of the surrogate mothers were less than 35 years old with the history of at least one normal pregnancy that was resulted to live healthy baby. Cryopreserved embryos were used in all of the patients. Endometrial preparation was similar in all surrogate mothers.

Estradiol vale rate (Estradiol vale rate, Aburaihan Co., Tehran, Iran) was started orally at the dose of 6 mg per day from the second day of menstrual cycle. Endometrial thickness was assessing by vaginal ultrasonography and when endometrial thickness reach more than 8 mm in diameter progesterone in oil (progesterone Aburaihan Co., Tehran, Iran) 100 mg IM daily was injected. Estradiol and progesterone administration was continued until the documentation of fetal heart activity by ultrasound. Thawing of the embryos was done two days after starting of progesterone injection. Embryos were transferred by using a Labotect (Labotect, Gottingen, Germany). Nine patients were studied and six of surrogate mothers developed a normal pregnancy (pregnancy rate=66.66%). No abortion was seen among these cases.

Case 1: a 34 year-old woman with history of ten years infertility and history of implantation failure for five times. 

Case 2: 32 years old with the history of infertility for ten years and five times implantation failure. Sonography showed pattern of adenomyosis of uterus. 

Case 3: 27 years old with the history of primary infertility for nine years and three times in vitro fertilization (IVF) that leads to early abortion. 

Case 4: 27 years old with the history of six years infertility and implantation failure for four times. Surrogate mother delivered healthy twin pregnancy.

Case 5: 39 years old with the history of infertility for eight years and three times history of repeated implantation failure. 

Case 6: 36 years old with the history of fourteen years infertility and six times IVF failure. Sonography showed adenomyosis. 

In all of these cases surrogacy resulted in pregnancy. No pregnancy was achieved in three others cases. 

Case 7: 36 years old with the history of primary infertility for seven years, RIF for three times. Myomectomy was done five years ago and the patient had no myoma during IVF. 

Case 8: 43 years old with the history of infertility for ten years and implantation failure for three times. 

Case 9: 35 years old with the history of infertility for fifteen years and history of implantation failure for three times. 

**Table I:** Basic characteristic of patients

**Variable**	**Minimum**	**Maximum**	**Mean ±SD**
Female age (years)	27	43	34.22 ± 4.99
Infertility duration (years)	3	15	8.33 ± 3.42
Basal FSH (mIU/ml)	4	10	6.88 ± 1.90
BMI (Kg/m^2^)	21	29	24.44 ± 2.60
**Etiology of infertility**	** Number (%)**		
Male factor	3 (33.33)		
Tubal factor	2 (22.22)		
Ovarian factor	2 (22.22)		
Unexplained	2 (22.22)		

## Discussion

In this case series we showed that surrogacy provide a good chance for pregnancy in patients with the history of RIF. We reported 66.66% pregnancy rate in nine patients. Three surrogate mother failed IVF again after use of surrogate uterus but one of the cases was a 43 year-old woman. Age is an independent factor for success of IVF and so failure of IVF in this case may be due to advance age of mother. Kling *et al* in their study for evaluation of pregnancy outcome after recurrent implantation failure reported 16.9% birth rate after fresh embryo transfer. In their study they showed that age is a prognostic factor for success in patient with RIF and pregnancy rate significantly decreased in women older than 39      (10). 

In case 3 the patient had a history of three time’s early abortion after embryo transfer. Recently it has been hypothesized that recurrent pregnancy loss and implantation failure have similar pathology and it was suggested that same factors that are important for fetal survival may by responsible for implantation failure (1, 11). According to this hypothesis we recommended surrogacy to the couple and fortunately achieved live birth. Two out of nine cases showed adenomyosis in sonography. 

Study demonstrated that adenomyosis is a potential cause in RIF and endometrial biopsy showed that adenomyosis was associated with increased aggregation of macrophages within superficial endometrial gland that can interfere with embryo implantation (12). Many maternal factors were suggested as etiology of RIF. However the role of these factors in the patients with RIF is a matter of debate and no agreement in the literature exist regarding effectiveness and necessity of evaluate of the factor in IVF outcomes. Besides sometimes no definitive etiology is found for RIF and no pregnancy achieve after treatments modality.

Endometrial receptivity now seems to be the restricted access of the implantation. Basic and clinical research will assist to progress understanding of the events of endometrial preparation for implantation. Microarray technology has spread vision, and has resulted in a number of gene expression analysis studies helped at translating answer into clinical application (13). This information could considerably improve the management of female infertility. More information is needed about molecular study and therapeutic options to treat endometrial dysfunction and improve implantation. Therefore it seems that elimination of endometrial factor by using surrogate mother may shed light on the unsolved question of the cases at unexplained RIF.
